# COVID-19 Vaccination Acceptance Among Chinese Population and Its Implications for the Pandemic: A National Cross-Sectional Study

**DOI:** 10.3389/fpubh.2022.796467

**Published:** 2022-02-08

**Authors:** Jian Wu, Mingze Ma, Yudong Miao, Beizhu Ye, Quanman Li, Clifford Silver Tarimo, Meiyun Wang, Jianqin Gu, Wei Wei, Lipei Zhao, Zihan Mu, Xiaoli Fu

**Affiliations:** ^1^Department of Health Management, College of Public Health, Zhengzhou University, Zhengzhou, China; ^2^Department of Science and Laboratory Technology, Dar es Salaam Institute of Technology, Dar es Salaam, Tanzania; ^3^Henan Provincial People's Hospital, People's Hospital of Zhengzhou University, Zhengzhou, China; ^4^School of Medicine, Southern University of Science and Technology, Shenzhen, China

**Keywords:** COVID-19, COVID-19 vaccine, vaccination, COVID-19 vaccination rate, China

## Abstract

**Objective:**

To examine the COVID-19 vaccination rate among a representative sample of adults from 31 provinces on the Chinese mainland and identify its influencing factors.

**Methods:**

We gathered sociodemographic information, data on people's awareness and behavior regarding COVID-19 and the COVID-19 vaccine, the accessibility of COVID-19 vaccination services, community environmental factors influencing people's awareness and behavior regarding the vaccination, information about people's skepticism on COVID-19 vaccine, and information about people's trust in doctors as well as vaccine developers through an online nationwide cross-sectional survey among Chinese adults (18 years and older). The odds ratios (OR) and 95% confidence intervals (CI) for the statistical associations were estimated using logistic regression models.

**Results:**

A total of 29,925 participants (51.4% females and 48.6% males) responded. 89.4% of the participants had already received a COVID-19 vaccination. After adjusting for demographic characteristics, awareness of COVID-19 pandemic/ COVID-19 vaccine, community environmental factors, awareness and behavior of general vaccinations, we discovered that having no religious affiliation, having the same occupational status as a result of coronavirus epidemic, being a non-smoker, always engaging in physical activity, having a lower social status, perceiving COVID-19 to be easily curable, and having easier access to vaccination are all associated with high vaccination rate (all *P* <0.05).

**Conclusions:**

31 provinces in mainland China currently have a relatively high rate of COVID-19 vaccination. To further increase the rate of COVID-19 vaccination, we must remove barriers associated with the community context and improve access to COVID-19 vaccine services. In addition, taking proactive and effective measures to address the reasons for non-vaccination with COVID-19 will aid in epidemic prevention and control.

## Introduction

Coronavirus disease 2019 (COVID-19), caused by severe acute respiratory syndrome coronavirus 2, emerged as a serious threat to human life at the end of 2019 and quickly spread to become a global pandemic, resulting in over 100 million illnesses and approximately one million deaths (1). Globally, weekly cases had been increasing for more than a month, with over 4 million cases reported in the past weeks and an average of over 570,000 cases were reported each day (2). As of August 4, 2021, there had been 93,374 confirmed cases and 4,636 deaths in mainland China (3). This epidemic has had a significant impact on social development and economic activity throughout the world, straining medical systems in numerous countries (1, 4). Personal preventive methods, including wearing masks and maintaining social distance, have been shown to be successful in slowing the spread of COVID-19, but may not completely eradicate the worldwide large-scale effects of COVID-19 (5–8).

Vaccination has evolved into a routine and highly effective method of illness prevention over the last century, significantly reducing or even eliminating certain viral diseases (9–11), and it is also one of the most effective public health interventions to date (12). Experts and researchers claim COVID-19 vaccination to be the most efficient way to contain this global pandemic (13, 14). Multiple pharmaceutical companies, scientific research and experimental institutes worldwide are rapidly designing, producing, and testing vaccines in response to the global consequences of COVID-19.

The population is vaccinated against COVID-19 using a variety of vaccines including mRNA vaccines (Pfizer, America), adenovirus vector vaccines (AstraZeneca, England), inactivated vaccines (CNBG, China), recombinant subunit vaccines (ZFSW, China), etc. (15, 16). Various companies have provided COVID-19 vaccines to countries around the world. As of August 4, 2021, more than 4 billion vaccination doses had been administered worldwide (17), with China accounting for 1.7 billion doses (18). Although the current data shows that the COVID-19 vaccine biotechnology is safe (1, 4, 19, 20), countries around the world are constantly boosting vaccine supply in order to improve vaccination accessibility. However, the global vaccination rate is still not high enough to build a population immunity barrier (2, 19). This situation exists as a result of major differences in vaccine supply around the globe (17), which contribute greatly to the disparity in vaccination rates between countries. In comparison to the vaccines for other infectious diseases, the COVID-19 vaccine was developed and approved for use in humans for a shorter period of time, as a result of which some people express hesitancy about vaccination (4, 21, 22). Concerns regarding vaccination safety are another factors that may affect people's decision to take a COVID-19 vaccine (23, 24).

However, studies indicate that depending exclusively on biotechnological advancements (effectiveness and safety) may be insufficient to further increase the COVID-19 vaccination rate in the population (4, 22). Thus, an unquantifiable gap may exist between the manufacturing and supply of the next round of global COVID-19 vaccination and its actual use. As a result, the risk of vaccine waste is increasing due to overproduction and investments in developing new vaccine manufacturing technology. It is hence critical to explore the actual factors that affect the coverage rate of the COVID-19 vaccine. In 2021, the COVID-19 epidemic occurred in some regions of China, including Guangzhou, Shenzhen, Nanjing, Chengdu, and Zhengzhou. Studies suggest that the severity of the COVID-19 outbreaks in various places, as well as the level of public awareness of the pandemic and the accessibility of the vaccination services may greatly influence the vaccination rates. However, it is still unclear how and to what extent these factors influenced COVID-19 vaccination rates in China mainland.

In the current study, we also assessed the community context, medical environment, and the acquisition of COVID-19 vaccinations as factors that may influence the vaccination rate. However, it is unclear if these factors influenced COVID-19 vaccination rates and the extent to which they did.

We recruited a saturated sample of participants from 31 provinces throughout mainland China and investigated the current COVID-19 vaccination rate while exploring its influencing factors. And further to provide effective analysis and suggestions for increasing the COVID-19 vaccination rate in China.

## Methods

### Participants and Procedures

**Figure 1:**
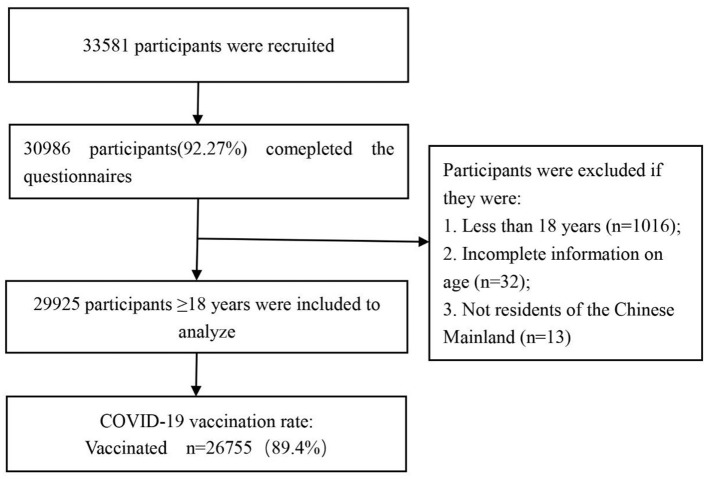
Flowchart of data process and analysis.

On July 10, 2021, we performed a preliminary online survey in Zhongmou County, Henan Province. Based on the COVID-19 vaccination rate and the reliability (α = 0.927) and validity (KMO = 0.966) of the questionnaire, we estimated the minimum sample size required for the formal survey to be 6,639 participants. This was likewise predicated on an 83.43% COVID-19 vaccination rate in the preliminary online survey, with a 1 percent acceptable error and taking into account the missing 20% sample size (detailed calculations are shown in Annex 1). A subsequent national cross-sectional online survey employing a snowball sampling approach among Chinese adults (≥18 years old) was conducted from 6th to 9th August 2021 by a market research company. During the study period, participants were unaware of the topic prior to their tentative consent to complete the survey. In order to ensure that the sample size for the current study was sufficient for estimating the vaccine coverage rate, sample saturation was monitored during the investigation (Annex 2). We ended the online survey when the sample reached 29,925 on 9th August 2021. Figure 1 depicts the flowchart for participant selection. The study protocol was approved by the Life Science Ethics Review Committee of the Zhengzhou University (Approval number: 2021-01-12-05).

### Assessments

The questionnaire used in the current study was designed based on the Oxford COVID-19 vaccine Hesitancy Scale, Vaccination knowledge Scale, Oxford Trust in Doctors and Developers Questionnaire, Vaccine Conspiracy Beliefs Scale (4), and the EuroQol Five Dimensions Questionnaire (5).

The questionnaire begins with a question on whether the respondent had ever received the primary COVID-19 vaccine. This question was designed to elicit information about vaccination rates whereby, item-specific response options coded ranging from 1 to 5 were used, including (1) Vaccinated, (2) Being vaccinated, (3) No, but prepared to receive the COVID-19 vaccine, (4) No, and I am not certain if I will receive the COVID-19 vaccine, and (5) No, I am hesitant to receive the COVID-19 vaccine. During the analysis, options (1) and (2) were merged into “Vaccinated” whereas options (3), (4), and (5) were merged into “Unvaccinated.”

Following that, our questionnaire elicited exploratory and confirmatory factors from five dimensions including; (1) Individual characteristics (i.e. sociodemographic data, subjective social status, and health status); (2) perceptions of COVID-19 pandemic progress (i.e. awareness of the global COVID-19 pandemic, local epidemic situation, and risk of COVID-19 infection); (3) perceptions of COVID-19 vaccine (i.e., trust in doctors and vaccine developers, skepticism in coronavirus and COVID-19 vaccine).

### Statistical Analysis

The Chi-square goodness-of-fit test was used to monitor sample saturation throughout the online survey (Annex 2). The Chi-square test was carried out to detect differences in COVID-19 vaccination rates across groups. Binary logistic regression was used to examine factors associated with COVID-19 vaccination coverage rate while adjusting for demographic and socio-economic confounders. All statistical analyses were carried out using SPSS version 21.0 and STATA version 16.0. *P*-value < 0.05 was considered statistically significant.

## Results

A total of 33,581 samples were gathered from 31 provinces on the Chinese mainland, with 30,986 respondents completing the survey. All participants filled out the form online. Following the deletion of ineligible samples in accordance with the inclusion and exclusion criteria, 29,925 samples remained. The flowchart of participant selection is presented in Figure 1.

### The COVID-19 Vaccination Rate in Mainland China

The sociodemographic characteristics, awareness level of the COVID-19 pandemic, awareness of the COVID-19 vaccine as well as COVID-19 vaccination coverage rate of the study participants are summarized in Table 1. All the results presented here are based on 29,925 valid questionnaires completed online in real time. Overall, 89.4% of participants (*n* = 26,755) had been vaccinated against COVID-19 whereas 10.6% (*n* = 3,170) had not. 51.4% of participants were female and the majority were non-religious (85%). The marital status of the individuals varied, with the majority being single (35.2%) or married (61.4%). 87.2% of the participants had completed at least a high school education whereas more than half of the participants did not change employment as a result of the coronavirus epidemic (56.1%). The COVID-19 vaccine hesitancy rate was 8.4%, with the majority of participants believing that their risk of novel coronavirus infection was either very low (65.6%) or moderate (15.5%), and 15.7% believing that they were at high risk of infection.

**Table 1:** Socio-demographic, awareness of COVID-19 pandemic, awareness of COVID-19 vaccine and COVID-19 vaccination rate information (*n* = 29,925).

**Variables**	**All participants (%)**	**Vaccination status**		
		**Vaccinated (%)**	**Unvaccinated (%)**	***P*** **-value**
**Total participants**	***n*** **=** **29,925 (100)**	***n*** **=** **26,755 (89.4)**	***n*** **=** **3,170 (10.6)**	
**Age ranges**				<0.001
18–29	13,312 (44.5)	11,694 (87.8)	1,618 (12.2)	
30–39	11,911 (39.8)	10,735 (90.1)	1,176 (9.9)	
40–49	3,269 (10.9)	3,044 (93.1)	225 (6.9)	
50–59	1,149 (3.8)	1,036 (90.2)	113 (9.8)	
≥60	284 (0.9)	246 (86.6)	38 (1.2)	
**Gender**				<0.001
Male	14,556 (48.6)	12,685 (87.1)	1,871 (12.9)	
Female	15,369 (51.4)	14,070 (91.5)	1,299 (8.5)	
**Marital status**				<0.001
Single	10,533 (35.2)	9,460 (89.8)	1,073 (10.2)	
Married	18,363 (61.4)	16,666 (90.8)	1,697 (9.2)	
Divorced	809 (2.7)	499 (61.7)	310 (38.3)	
Widowed	178 (0.6)	99 (55.6)	79 (44.4)	
Others	42 (0.1)	31 (73.8)	11 (26.2)	
**Religious faith**				<0.001
None	25,424 (85.0)	23,328 (91.8)	2,196 (8.2)	
Buddhism	2,720 (9.1)	2,299 (84.5)	421 (15.5)	
Christian	1,122 (3.7)	770 (68.6)	352 (31.4)	
Taoism	401 (1.3)	254 (63.3)	147 (36.7)	
Mohammedanism	142 (0.5)	106 (74.6)	36 (25.4)	
Others	116 (0.4)	98 (84.5)	18 (15.5)	
**Education status**				<0.001
Illiteracy	257 (0.9)	167 (65)	90 (35)	
Primary school and below	891 (3.0)	594 (66.7)	297 (33.3)	
Junior high school	2,691 (9.0)	2,187 (81.3)	504 (18.7)	
Senior high school	7,893 (26.4)	7,032 (89.1)	861 (10.9)	
College degree and above	18,193 (60.8)	16,775 (92.2)	1,418 (7.8)	
**Employment change due to coronavirus**				<0.001
None	16,784 (56.1)	15,610 (93.0)	1,174 (7.0)	
None, but working from a different location (e.g., work from home)	4,238 (14.2)	3,631 (85.7)	607 (14.3)	
Working hours have reduced	4,777 (16.0)	4,103 (85.9)	674 (14.1)	
Working hours have increased	2,320 (7.8)	1,942 (83.7)	378 (16.3)	
Unemployed	1,037 (3.5)	806 (77.7)	231 (22.3)	
Newly employed (full-time)	500 (1.7)	429 (85.8)	71 (14.2)	
Newly employed (part-time)	269 (0.9)	234 (87.0)	35 (13.0)	
**Smoking status**				<0.001
Non-smoker	18,559 (62.0)	17,510 (94.3)	1,049 (5.7)	
Smoker	9,702 (32.4)	7,763 (80.0)	1,939 (20.0)	
Ever-smoker	1,664 (5.6)	1,482 (89.1)	182 (10.9)	
**Drinking status**				<0.001
Non-drinker	10,361 (34.6)	9,703 (93.6)	658 (6.4)	
Drinker	18,484 (61.8)	16,122 (87.2)	2,362 (12.8)	
Ever-drinker	1,080 (3.6)	930 (86.1)	150 (13.9)	
**Physical activity**				<0.001
Always	9,912 (33.1)	9,233 (93.1)	679 (6.9)	
Often	8,579 (28.7)	7,694 (89.7)	885 (10.3)	
Sometimes	7,451 (24.9)	6,413 (86.1)	1,038 (13.9)	
Seldom	3,346 (11.2)	2,887 (86.3)	459 (13.7)	
Never	637 (2.1)	528 (82.9)	109 (17.1)	
**Physical examination**				<0.05
Yes	28,797 (96.2)	25,768 (89.5)	3,029 (10.5)	
No	1,128 (3.8)	987 (87.5)	141 (12.5)	
**Family doctor**				<0.001
Yes	9,654 (32.3)	8,473 (87.8)	1,181 (12.2)	
No	19,127 (63.9)	17,314 (90.5)	1,813 (9.5)	
Do not know	1,144 (3.8)	968 (84.6)	176 (15.4)	
**Self-assessment of health status (EQ-5D)**				<0.001
Level 1	7,534 (25.2)	6,112 (81.1)	1,422 (18.9)	
Level 2	7,982 (26.7)	7,286 (91.3)	696 (8.7)	
Level 3	7,183 (24.0)	6,707 (93.4)	476 (6.6)	
Level 4	7,226 (24.1)	6,650 (92.0)	576 (8.0)	
**Subjective social status in the community**				<0.001
Level 1	8,193 (27.4)	7,524 (91.8)	669 (8.2)	
Level 2	8,311 (27.8)	7,407 (89.1)	904 (10.9)	
Level 3	8,926 (29.8)	7,767 (87.0)	1,159 (13.0)	
Level 4	4,495 (15.0)	4,057 (90.3)	438 (9.7)	
**Subjective social status in society**				<0.001
Level 1	9,419 (31.5)	8,617 (91.5)	802 (8.5)	
Level 2	9,751 (32.6)	8,612 (88.3)	1,139 (11.7)	
Level 3	4,689 (15.7)	4,127 (88.0)	562 (12.0)	
Level 4	6,066 (20.3)	5,399 (89.0)	667 (11.0)	
**Access to COVID-19 vaccine information**				<0.001
Community worker	8,416 (28.1)	7,872 (93.5)	544 (6.5)	
Internet (such as WeChat, Douyin, Weibo, etc.)	15,522 (51.9)	13,921 (89.7)	1,601 (10.3)	
Family, relatives, friends, neighbors, etc.	1,718 (5.7)	1,289 (75.0)	429 (25.0)	
Television	1,258 (4.2)	1,006 (80.0)	252 (20.0)	
Medical servant	1,564 (5.2)	1,389 (88.8)	175 (11.2)	
Newspapers, magazines, leaflets, etc.	420 (1.4)	346 (82.4)	74 (17.6)	
Vaccine related lectures and seminars	901 (3.0)	826 (91.7)	75 (8.3)	
Others	126 (0.4)	106 (84.1)	20 (15.9)	
**Awareness of COVID-19 pandemic**				
**Views on the global COVID-19 outbreak**				<0.001
Serious	26,339 (88.0)	24,213 (91.9)	2,126 (8.1)	
General	2,215 (7.4)	1,531 (69.1)	684 (30.9)	
Not serious	1,077 (3.6)	770 (71.5)	307 (28.5)	
Do not know	294 (1.0)	241 (82.0)	53 (18.0)	
**Views on the COVID-19 outbreak in own current place of residence**				<0.001
Serious	6,570 (22.0)	5,470 (83.3)	1,100 (16.7)	
General	6,577 (22.0)	5,645 (85.8)	932 (14.2)	
Not serious	16,436 (54.9)	15,362 (93.5)	1,074 (6.5)	
Do not know	342 (1.1)	278 (81.3)	64 (18.7)	
**Risk of COVID-19 infection**				<0.001
High risk	4,708 (15.7)	3,781 (80.3)	927 (19.7)	
Moderate risk	4,636 (15.5)	3,889 (83.9)	747 (16.1)	
Low risk	19,638 (65.6)	18,275 (93.1)	1,363 (6.9)	
Do not know	943 (3.2)	810 (85.9)	133 (14.1)	
**Curability of COVID-19**				<0.001
Possible	23,527 (78.6)	21,703 (92.2)	1,824 (7.8)	
Do not sure	3,625 (12.1)	2,803 (77.3)	822 (22.7)	
Impossible	2,044 (6.8)	1,632 (79.8)	412 (20.2)	
Do not know	729 (2.4)	617 (84.6)	112 (15.4)	
**Awareness of COVID-19 vaccine**				
**Views on the effectiveness of vaccination against COVID-19**				<0.001
Effective	25,941 (86.7)	24,034 (92.6)	1,907 (7.4)	
Not sure	2,979 (10.0)	2,141 (71.9)	838 (28.1)	
Invalid	768 (2.6)	406 (52.9)	362 (47.1)	
Do not know	237 (0.8)	174 (73.4)	63 (26.6)	
**Views on the necessary for all people to be vaccinated**				<0.001
Necessary	27,370 (91.5)	25,434 (92.9)	1,936 (7.1)	
Not sure	1,883 (6.3)	1,018 (54.1)	865 (45.9)	
Not necessary	672 (2.2)	303 (45.1)	369 (54.9)	
**Setting up a special COVID-19 vaccination site in the community**				<0.001
Necessary	27,987 (93.5)	25,621 (91.5)	2,366 (8.5)	
Not sure	1,429 (4.8)	851 (59.6)	578 (40.4)	
Not necessary	509 (1.7)	283 (55.6)	226 (44.4)	
**Willing to get vaccinated against COVID-19**				<0.001
Accept	27,411 (91.6)	25,552 (93.2)	1,859 (6.8)	
Delay	1,860 (6.2)	935 (50.3)	925 (49.7)	
Refuse	654 (2.2)	268 (41.0)	386 (59.0)	
**The availability of vaccination in the community**				<0.001
Convenient	28,164 (94.1)	25,559 (90.8)	2,605 (9.2)	
General	1,401 (4.7)	969 (69.2)	432 (30.8)	
Not convenient	360 (1.2)	227 (63.1)	133 (36.9)	

The study also indicates that women vaccinated against COVID-19 at a higher rate (52.6%) compared to men (47.4%).Vaccination acceptance was found to be associated with participants age (18–39), female gender, married status, having no religious affiliation, being a graduate of high school and above, maintaining occupational status following the epidemic, being non-smoker, being physically active, physical examination, and having lower social status in the community, to mention a few. Additional participant characteristics are shown in Table 1.

**Figure 2:**
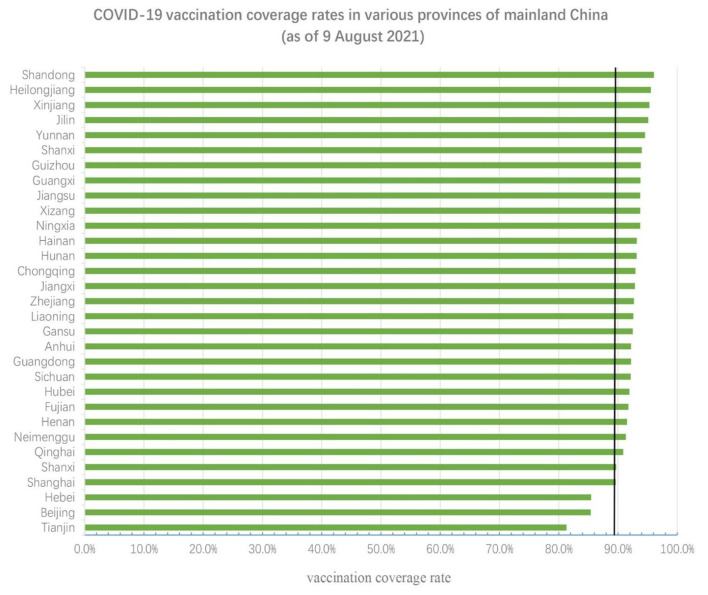
COVID-19 vaccination rates in different provinces. The black vertical line represents the national average level (89.4%).

As illustrated in Figure 2, the COVID-19 vaccination rate in all provinces in mainland China exceeded 81.3%, with Shandong province having the highest rate of vaccination at 96%. Additional detailed data is presented in Figure 2.

### Factors Affecting the Rate of COVID-19 Vaccination

The logistic regression model showed that age, marital status, religion, employment change due to coronavirus, smoking status, physical activity, self-assessment of health status (EQ-5D), subjective social status in the community, information source for COVID-19 vaccine, the awareness of COVID-19 pandemic and the awareness of COVID-19 vaccine were found to be independently associated with COVID-19 vaccination coverage rate. Being married, non-smoker and willing to get vaccinated, were all positively associated with vaccination rate. Detailed results are shown in Table 2.

**Table 2:** Associations of demographic factors, awareness of COVID-19 pandemic, awareness of COVID-19 vaccine with COVID-19 vaccination rate (individual regressions) (*n* = 29,925).

**Covariates**	**OR (95% CI)**	***P*** **-value**	**Adjust OR (95% CI)**	***P*** **-value**
**Demographic characteristics**				
**Age**				
18–29	ref.	ref.	ref.	ref.
30–39	1.26 (1.17–1.37)	<0.001	0.99 (0.90–1.10)	0.896
40–49	1.87 (1.62–2.16)	<0.001	1.35 (1.13–1.60)	0.001
50–59	1.27 (1.04–1.55)	0.020	1.10 (0.86–1.40)	0.452
60	0.90 (0.63–1.27)	0.532	1.52 (0.95–2.43)	0.079
**Gender**				
Male	ref.	ref.	ref.	ref.
Female	0.63 (0.58–0.67)	<0.001	0.99 (0.91–1.09)	0.865
**Marital status**				
Single	ref.	ref.	ref.	ref.
Married	0.90 (0.83–0.97)	0.009	1.14 (1.02–1.27)	0.017
Divorced	5.48 (4.69–6.40)	<0.001	1.32 (1.07–1.64)	0.011
Widowed	7.03 (5.19–9.52)	<0.001	1.47 (0.98–2.20)	0.063
Others	3.12 (1.56–6.24)	0.001	1.90 (0.81–4.45)	0.141
**Religious faith**				
None	ref.	ref.	ref.	ref.
Buddhism	1.94 (1.73–2.16)	<0.001	1.05 (0.91–1.20)	0.537
Christian	4.84 (4.23–5.53)	<0.001	1.36 (1.14–1.64)	0.001
Taoism	6.12 (4.97–7.53)	<0.001	1.25 (0.94–1.66)	0.119
Mohammedanism	3.59 (2.45–5.26)	<0.001	0.93 (0.56–1.52)	0.761
Others	1.94 (1.17–3.21)	0.010	1.39 (0.77–2.55)	0.275
**Education status**				
Illiteracy	ref.	ref.	ref.	ref.
Primary school and below	0.93 (0.69–1.24)	0.614	1.28 (0.88–1.84)	0.193
Junior high school	0.43 (0.33–0.56)	<0.001	0.97 (0.68–1.38)	0.864
Senior high school	0.23 (0.17–0.30)	<0.001	0.88 (0.63–1.24)	0.466
College degree and above	0.16 (0.12–0.20)	<0.001	0.75 (0.53–1.05)	0.092
**Employment change due to coronavirus**				
None	ref.	ref.	ref.	ref.
None, but working from a different location (e.g., work from home)	2.22 (2.00–2.47)	<0.001	1.26 (1.11–1.42)	0.000
Working hours have reduced	2.18 (1.97–2.42)	<0.001	1.23 (1.09–1.39)	0.001
Working hours have increased	2.59 (2.28–2.93)	<0.001	1.28 (1.09–1.49)	0.001
Unemployed	3.81 (3.25–4.46)	<0.001	1.47 (1.20–1.79)	0.000
Newly employed (full-time)	2.20 (1.70–2.85)	<0.001	1.14 (0.83–1.55)	0.439
Newly employed (part-time)	1.99 (1.39–2.85)	<0.001	1.20 (0.78–1.85)	0.417
**Smoking status**				
Non-smoker	ref.	ref.	ref.	ref.
Smoker	4.17 (3.85–4.51)	<0.001	1.74 (1.56–1.94)	0.000
Ever-smoker	2.04 (1.74–2.42)	<0.001	1.18 (0.96–1.47)	0.122
**Drinking status**				
Non-drinker	ref.	ref.	ref.	ref.
Drinker	2.16 (1.97–2.36)	<0.001	1.04 (0.93–1.16)	0.496
Ever-drinker	2.38 (1.97–2.87)	<0.001	1.00 (0.78–1.28)	0.998
**Physical activity**				
Always	ref.	ref.	ref.	ref.
Often	1.56 (1.40–1.74)	<0.001	1.15 (1.02–1.30)	0.022
Sometimes	2.20 (1.98–2.44)	<0.001	1.44 (1.28–1.63)	0.000
Seldom	2.16 (1.91–2.45)	<0.001	1.46 (1.26–1.71)	0.000
Never	2.80 (2.25–3.50)	<0.001	1.26 (0.95–1.66)	0.109
**Physical examination**				
Yes	ref.	ref.	ref.	ref.
No	1.22 (1.01–1.46)	0.034	0.83 (0.67–1.04)	0.113
**Family doctor**				
Yes	ref.	ref.	ref.	ref.
No	0.75 (0.70–0.81)	<0.001	0.96 (0.87–1.06)	0.384
Do not know	1.30 (1.10–1.55)	0.002	1.00 (0.80–1.24)	0.999
**Self-assessment of health status (EQ-5D)**				
Level 1	ref.	ref.	ref.	ref.
Level 2	0.41 (0.37–0.45)	<0.001	0.79 (0.71–0.89)	<0.001
Level 3	0.31 (0.27–0.34)	<0.001	0.67 (0.59–0.76)	<0.001
Level 4	0.37 (0.34–0.41)	<0.001	0.71 (0.62–0.80)	<0.001
**Subjective social status in the community**				
Level 1	ref.	ref.	ref.	ref.
Level 2	1.37 (1.24–1.52)	<0.001	1.18 (1.04–1.34)	0.012
Level 3	1.68 (1.52–1.86)	<0.001	1.19 (1.02–1.38)	0.022
Level 4	1.21 (1.07–1.38)	0.003	1.04 (0.85–1.28)	0.710
**Subjective social status in society**				
Level 1	ref.	ref.	ref.	ref.
Level 2	1.42 (1.29–1.56)	<0.001	1.17 (1.03–1.32)	0.012
Level 3	1.46 (1.31–1.64)	<0.001	1.10 (0.937–1.30)	0.241
Level 4	1.33 (1.19–1.48)	<0.001	1.08 (0.90–1.30)	0.385
**Access to COVID-19 vaccine information**				
Community worker	ref.	ref.	ref.	ref.
Internet (such as WeChat, Douyin, Weibo, etc.)	1.66 (1.50–1.84)	<0.001	0.36 (1.21–1.52)	0.000
Family, relatives, friends, neighbors, etc.	4.82 (4.19–5.54)	<0.001	1.53 (1.29–1.82)	0.000
Television	3.62 (3.08–4.27)	<0.001	1.47 (1.20–1.80)	0.000
Medical servant	1.82 (1.52–2.18)	<0.001	1.20 (0.97–1.48)	0.092
Newspapers, magazines, leaflets, etc.	3.09 (2.37–4.04)	<0.001	1.23 (0.88–1.72)	0.028
Vaccine related lectures and seminars	1.31 (1.02–1.69)	0.034	0.94 (0.70–1.28)	0.720
Others	2.73 (1.68–4.44)	<0.001	1.21 (0.70–2.10)	0.493
**Awareness of COVID-19 pandemic**				
**Views on the global COVID-19 outbreak**				
Serious	ref.	ref.	ref.	ref.
General	5.08 (4.06–5.63)	<0.001	1.52 (1.33–1.73)	0.000
Not serious	4.54 (3.95–5.22)	<0.001	1.31 (1.08–1.59)	0.005
Do not know	2.50 (1.85–3.38)	<0.001	0.83 (0.56–1.23)	0.360
**Views on the COVID-19 outbreak in own current place of residence**				
Serious	ref.	ref.	ref.	ref.
General	0.82 (0.75–0.90)	<0.001	0.85 (0.75–0.95)	0.005
Not serious	0.35 (0.32–0.38)	<0.001	0.60 (0.53–0.67)	<0.001
Do not know	1.14 (0.87–1.51)	0.343	0.44 (0.30–0.65)	<0.001
**Risk of COVID-19 infection**				
High risk	ref.	ref.	ref.	ref.
Moderate risk	0.78 (0.70–0.87)	<0.001	0.77 (0.68–0.88)	<0.001
Low risk	0.30 (0.28–0.33)	<0.001	0.64 (0.57–0.72)	<0.001
Do not know	0.67 (0.55–0.82)	<0.001	0.56 (0.43–0.74)	<0.001
**Possibility of cure**				
Possible	ref.	ref.	ref.	ref.
Do not sure	3.48 (3.18–3.82)	<0.001	1.61 (1.44–1.80)	<0.001
Impossible	3.00 (2.67–3.38)	<0.001	1.57 (1.36–1.82)	<0.001
Do not know	2.15 (1.76–2.66)	<0.001	1.37 (1.05–1.77)	0.019
**Awareness of COVID-19 vaccine**				
**Views on the effectiveness of vaccination against COVID-19**				
Effective	ref.	ref.	ref.	ref.
Not sure	4.93 (4.50–5.41)	<0.001	1.89 (1.69–2.12)	<0.001
Invalid	11.24 (9.68–13.04)	<0.001	1.68 (1.39–2.03)	<0.001
Do not know	4.56 (3.41–6.11)	<0.001	1.44 (0.99–2.10)	0.059
**Views on the necessary for all people to be vaccinated**				
Necessary	ref.	ref.	ref.	ref.
Not sure	11.16 (10.08–12.36)	<0.001	3.37 (2.98–3.82)	<0.001
Not necessary	15.99 (13.65–18.75)	<0.001	3.13 (2.57–3.81)	<0.001
**Setting up a special COVID-19 vaccination site in the community**				
Necessary	ref.	ref.	ref.	ref.
Not sure	7.35 (6.56–8.24)	<0.001	1.47 (1.27–1.70)	<0.001
Not necessary	8.65 (7.22–10.35)	<0.001	1.11 (0.87–1.41)	0.403
**Willing to get vaccinated against COVID-19**				
Accept	ref.	ref.	ref.	ref.
Delay	13.60 (12.27–15.06)	<0.001	4.02 (3.56–4.54)	<0.001
Refuse	19.80 (16.82–23.29)	<0.001	4.29 (3.54–5.20)	<0.001
**The availability of vaccination in the community**				
Convenient	ref.	ref.	ref.	ref.
General	4.37 (3.88–4.93)	<0.001	1.84 (1.58–2.15)	<0.001
Not convenient	5.75 (4.62–7.15)	<0.001	2.09 (1.57–2.78)	<0.001

*OR, odds ratio; CI, confidence intervals*.

*Levels 1–4: indicate progressively higher scores. Lower levels indicating poorer self-assessment of health status and lower subjective social status in the community and society*.

*Adjust OR (95% CI): adjusted age, gender, marital status, religious faith, educational status, employment change due to coronavirus, smoking status, drinking status, physical activity, physical examination, family doctor, self-assessment of health status (EQ-5D), subjective social status in the community/society, access to COVID-19 vaccine information, views on the global/local COVID-19 outbreak, risk of COVID-19 infection, curability of COVID-19, the effectiveness of vaccination, the necessary for all people to be vaccinated, the need for special vaccination sites and the accessibility of vaccination in communities*.

Additionally, as shown in Table 3, we discovered that contextual factors such as community environmental factors, vaccine perception and behavior influence COVID-19 vaccination uptake. The binary logistic regression model indicates that having easier access to vaccination, sharing similar views on COVID-19 vaccination with other residents, being willing to discuss vaccine issues with other residents, setting up vaccination sites, the good performance of community leaders, residents' concern for one another, hope to live in their community for a long time, and perceptions of generic vaccines, safety, and effectiveness are also independently associated with COVID-19 vaccination.

**Table 3:** Association between background information and COVID-19 vaccination rate (*n* = 29,925).

**Covariates**	**Vaccinated**	**Unvaccinated**	***P*** **-value**	**OR (95% CI)**	***P*** **-value**	**Adjust OR (95% CI)**	***P*** **-value**
	26,755 (89.4)	3,170 (10.6)					
**Community environmental factors**							
**The availability of vaccination in the community**							
Convenient	25,559 (95.5)	2,605 (82.2)	<0.001	ref.	ref.	ref.	ref.
General	969 (3.6)	432 (13.6)	<0.001	4.37 (3.88–4.93)	<0.001	1.84 (1.60–2.12)	<0.001
Not convenient	227 (0.8)	133 (4.2)	<0.001	5.75 (4.62–7.15)	<0.001	1.97 (1.52–2.54)	<0.001
**Have the same views as other residents in this community on COVID-19 vaccination**							
Same	22,114 (82.7)	2,013 (63.5)	<0.001	ref.	ref.	ref.	ref.
Not sure	3,602 (13.5)	823 (26.0)	<0.001	2.51 (2.29–2.74)	<0.001	1.24 (1.11–1.37)	<0.001
Different	1,039 (3.9)	334 (10.5)	<0.001	3.53 (3.09–4.03)	<0.001	1.54 (1.32–1.81)	<0.001
**Willing to discuss COVID-19 vaccine-related issues with other residents of this community**							
Yes	24,619 (92.0)	2,241 (70.7)	<0.001	ref.	ref.	ref.	ref.
Not sure	1,601 (6.0)	655 (20.7)	<0.001	4.50 (4.06–4.97)	<0.001	1.55 (1.37–1.75)	<0.001
No	535 (2.0)	274 (8.6)	<0.001	5.63 (4.83–6.55)	<0.001	1.57 (1.29–1.89)	<0.001
**Setting up a special COVID-19 vaccination site in the community**							
Necessary	25,621 (95.8)	2,366 (74.6)	<0.001	ref.	ref.	ref.	ref.
Not sure	851 (3.2)	578 (18.2)	<0.001	7.35 (6.56–8.24)	<0.001	2.39 (2.08–2.73)	<0.001
Not necessary	283 (1.1)	226 (7.1)	<0.001	8.65 (7.22–10.35)	<0.001	2.27 (1.83–2.81)	<0.001
**The performance of the community's leaders in the COVID-19 vaccination**							
Well done	24,881 (93.0)	2,269 (71.6)	<0.001	ref.	ref.	ref.	ref.
General	1,595 (6.0)	695 (21.9)	<0.001	4.79 (4.32–5.27)	<0.001	1.73 (1.52–19.5)	<0.001
Not good	279 (1.0)	206 (6.5)	<0.001	8.10 (6.73–9.74)	<0.001	1.65 (1.32–2.06)	<0.001
**Caring about what other residents in the community think of themselves**							
Care about	16,880 (63.1)	1,791 (56.5)	<0.001	ref.	ref.	ref.	ref.
General	7,011 (26.2)	968 (30.5)	<0.001	1.30 (1.20–1.41)	<0.001	0.89 (0.81–0.98)	0.014
Not care about	1,864 (10.7)	411 (13.0)	<0.001	1.35 (1.21–1.52)	<0.001	0.87 (0.75–0.98)	0.029
**Residents in this community care for and help each other**							
Agree	22,956 (85.8)	1,996 (63.0)	<0.001	ref.	ref.	ref.	ref.
General	3,332 (12.5)	866 (27.3)	<0.001	2.99 (2.74–3.26)	<0.001	1.46 (1.32–1.63)	<0.001
Do not agree	467 (1.7)	308 (9.7)	<0.001	7.59 (6.52–8.82)	<0.001	1.84 (1.53–2.22)	<0.001
**Hope to live in this community for a long time**							
Agree	23,084 (86.3)	2,062 (65.0)	<0.001	ref.	ref.	ref.	ref.
General	2,970 (11.1)	836 (26.4)	<0.001	3.15 (2.88–3.44)	<0.001	1.44 (1.30–1.68)	<0.001
Do not agree	701 (2.6)	272 (8.6)	<0.001	4.34 (3.75–5.03)	<0.001	1.26 (1.06–1.52)	0.011
**Awareness and behavior of general vaccinations**							
**Awareness of general vaccinations**							
Level 1	7,351 (27.5)	1,291 (40.7)	<0.001	ref.	ref.	ref.	ref.
Level 2	5,443 (20.3)	1,060 (33.4)	<0.001	1.11 (1.01–1.21)	0.022	1.14 (1.03–1.25)	0.010
Level 3	8,947 (33.4)	646 (20.4)	<0.001	0.41 (0.37–0.45)	<0.001	0.70 (0.63–0.79)	<0.001
Level 4	5,014 (18.7)	173 (5.5)	<0.001	0.20 (0.17–0.23)	<0.001	0.47 (0.40–0.56)	<0.001
**Concerns about the safety risks of general vaccinations**							
Worried	7,939 (29.7)	1,614 (50.9)	<0.001	ref.	ref.	ref.	ref.
General	6,081 (22.7)	865 (27.3)	<0.001	0.70 (0.64–0.76)	<0.001	0.68 (0.61–0.76)	<0.001
Do not worried	12,735 (47.6)	691 (21.8)	<0.001	0.27 (0.24–029)	<0.001	0.47 (0.41–0.53)	<0.001
**Concerns about the effectiveness of general vaccinations**							
Worried	8,957 (33.5)	1,524 (48.1)	<0.001	ref.	ref.	ref.	ref.
General	5,683 (21.2)	938 (29.6)	<0.001	0.97 (0.89–1.05)	0.498	1.07 (0.96–1.19)	0.195
Do not worried	12,115 (45.3)	708 (22.3)	<0.001	0.34 (0.31–0.38)	<0.001	0.80 (0.70–0.91)	0.001
**Have received general vaccinations**							
Yes	22,827 (85.3)	1,839 (58.0)	<0.001	ref.	ref.	ref.	ref.
No	2,657 (9.9)	1,030 (32.5)	<0.001	4.81 (4.41–5.25)	<0.001	2.54 (2.30–2.80)	<0.001
Do not clear	1,271 (4.8)	301 (9.5)	<0.001	2.94 (2.57–3.36)	<0.001	1.46 (1.25–1.70)	<0.001

*OR, odds ratio; CI, confidence intervals*.

*Levels 1–4: indicate progressively higher scores. Lower levels indicating poorer awareness of general vaccines*.

*Adjust OR (95% CI): adjusted the convenience of community vaccination, the same views as other residents on COVID-19 vaccination, the willingness to discuss vaccine issues with other residents, setting up vaccination sites, the performance of community leaders, caring about other residents' opinions, residents caring for each other, hope to live in this community for a long time, perceptions of generic vaccines, safety, effectiveness, and whether had received generic vaccines*.

In addition, low level of coronavirus skepticism, COVID-19 vaccine skepticism and trust in healthcare system are also positively associated with COVID-19 vaccination. Detailed results are presented in Table 4.

**Table 4:** Associations of coronavirus skepticism, COVID-19 vaccine skepticism, trust in healthcare system with COVID-19 vaccination coverage rate.

**Covariates**	**Vaccinated**	**Unvaccinated**	***P*** **-value**	**OR (95% CI)**	***P*** **-value**	**Adjust OR (95% CI)**	***P*** **-value**
**Coronavirus skepticism**							
Level 1	6,570 (24.6)	1,046 (33.0)	<0.001	ref.	ref.	ref.	ref.
Level 2	6,514 (24.3)	1,314 (41.5)	<0.001	1.28 (1.16–1.38)	<0.001	1.71 (1.55–1.88)	<0.001
Level 3	6,910 (25.8)	596 (18.8)	<0.001	0.54 (0.49–0.60)	<0.001	1.22 (1.07–1.38)	0.002
Level 4	6,761 (25.3)	214 (6.8)	<0.001	0.20 (0.17–0.23)	<0.001	0.80 (0.67–0.97)	0.023
**COVID-19 vaccine skepticism**							
Level 1	6,231 (23.3)	1,497 (47.2)	<0.001	ref.	ref.	ref.	ref.
Level 2	9,068 (33.9)	1,252 (39.5)	<0.001	0.57 (0.53–0.62)	<0.001	0.81 (0.74–0.90)	<0.001
Level 3	4,386 (16.4)	238 (7.5)	<0.001	0.23 (0.20–0.26)	<0.001	0.43 (0.37–0.51)	<0.001
Level 4	7,070 (26.4)	183 (5.8)	<0.001	0.11 (0.09–0.13)	<0.001	0.22 (0.18–0.27)	<0.001
**Trust in medical staff**							
Trust	22,125 (82.7)	1,521 (48.0)	<0.001	ref.	ref.	ref.	ref.
General	2,067 (7.7)	528 (16.7)	<0.001	3.72 (3.33–4.14)	<0.001	2.22 (1.98–2.49)	<0.001
Do not trust	2,563 (9.6)	1,121 (35.4)	<0.001	6.36 (5.83–6.94)	<0.001	4.41 (4.02–4.84)	<0.001
**Trust in vaccine developers**							
Trust	20,183 (75.4)	1,642 (51.8)	<0.001	ref.	ref.	ref.	ref.
General	3,507 (13.1)	874 (27.6)	<0.001	3.06 (2.80–3.35)	<0.001	1.44 (1.30–1.58)	<0.001
Do not trust	3,065 (11.5)	654 (20.6)	<0.001	2.62 (2.38–2.89)	<0.001	1.86 (1.67–2.06)	<0.001

*OR, odds ratio; CI, confidence intervals*.

*Levels 1–4: indicate progressively higher scores. Lower levels indicating higher levels of suspicion*.

*Adjust OR (95% CI):adjusted coronavirus skepticism, COVID-19 vaccine skepticism, trust in medical staff, trust in vaccine developers*.

### Analysis of the Reasons for Non-vaccination Against COVID-19

**Figure 3:**
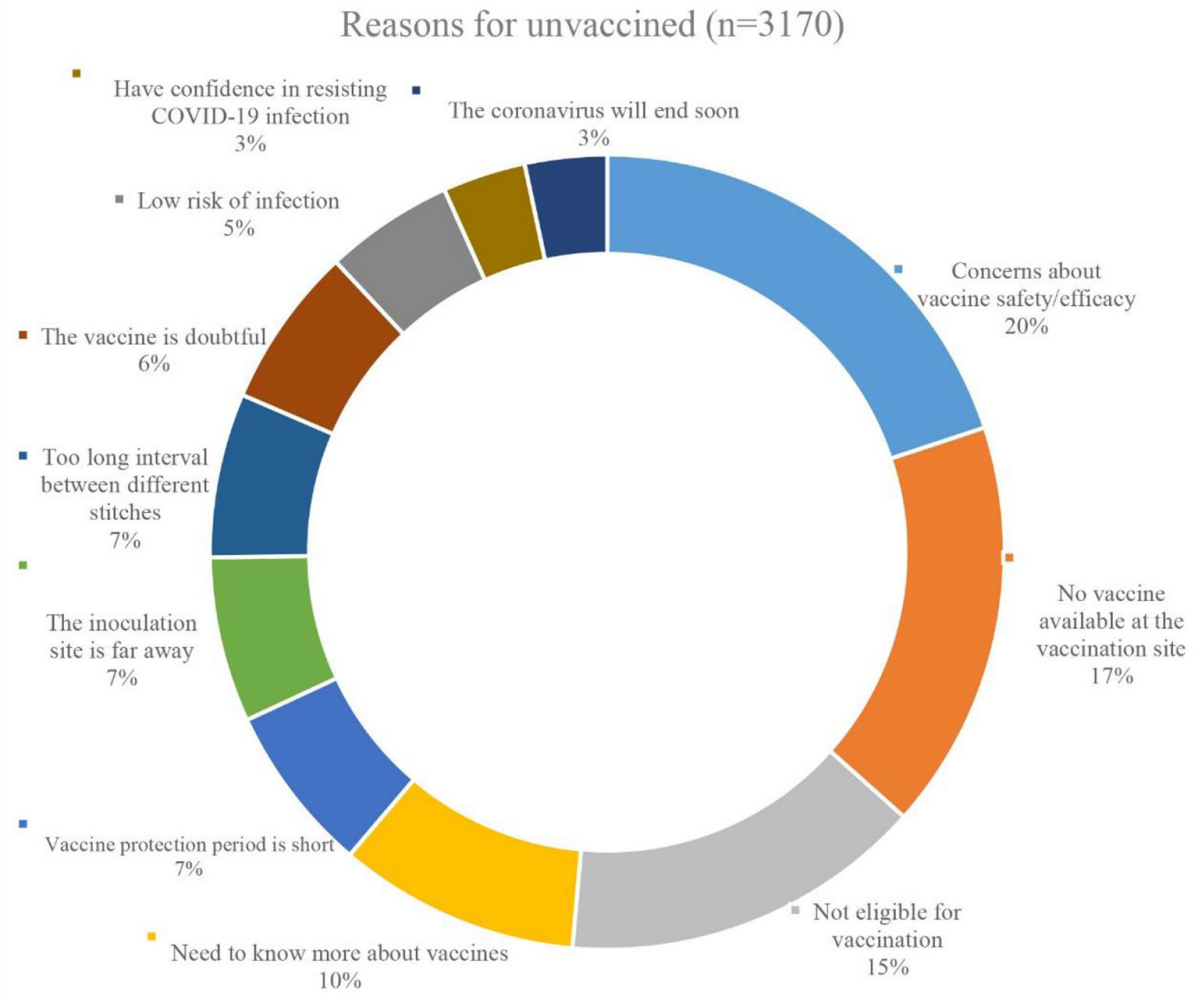
The reasons for unvaccinated (*n* = 3,170).

Three thousand one hundred and seventy participants were unvaccinated against COVID-19 out of a total of 29,925. As a result, we conducted research and analysis on the factors that contributed to this portion of the individuals being unvaccinated. As demonstrated in Figure 3, the most frequently cited reasons for non-vaccination were specific concerns about the COVID-19 vaccine's safety or effectiveness (20%), or a request for additional vaccine information (10%). 17% of those who were not vaccinated cited a lack of vaccine at the vaccination location, while 15% were not vaccinated owing to a medical condition that precluded them from obtaining COVID-19 vaccine. Figure 3 illustrates the remaining causes for non-vaccination.

## Discussion

COVID-19 vaccination has emerged as a critical component of the global public health response to the severe acute respiratory syndrome coronavirus 2 (SARS-CoV-2) pandemic (11, 13, 25). As a result, the World Health Organization (WHO) is dedicated to urging countries worldwide to boost COVID-19 vaccine coverage and to collaborating on the development of an effective epidemic prevention and control mechanism (12, 17). China may be a world leader in COVID-19 vaccination coverage. This study examined COVID-19 vaccination in a large representative sample of 31 provinces in mainland China. As of August 4, 2021, the majority of participants (89.4%) had received COVID-19 vaccinations, while just a minority (10.6%) had not. This ratio is likely to stay high throughout the impending COVID-19 vaccination booster, although further investigation and confirmation are necessary. The high COVID-19 vaccination rate in China mainland may be attributable to the following factors: First, China has formulated a vaccine management law and successfully passed the WHO evaluation of its National Vaccine Management System (NRS) which guarantees the quality and supply of vaccines (26, 27). Second, the Chinese government has continued to increase post-marketing surveillance of vaccinations, placing a priority on vaccine safety and effectiveness, and has a continuous track record of vaccine-preventable diseases and public acceptance of vaccines. Additionally, vaccination usage experience and big data on vaccines are being developed and tracked (28). Third, China has strengthened the risk communication, increased publicity to inform recipients as well as the public about the benefits and risks of vaccination, and actively promoted the scientific premise that the overall benefits of vaccines outweigh the risks. Finally, China is committed to expanding the availability of vaccines by setting up temporary vaccination sites according to the characteristics of the jurisdiction and population, which greatly facilitates the vaccination of residents.

The global outbreak of the epidemic will lead to an increase in the demand of people for COVID-19 vaccine, which will in turn increase its vaccination rate (1, 29, 30). Table 2 indicates that people's awareness of COVID-19 had an obvious effect on vaccination rate. The global epidemic outbreak, the evolution of the epidemic situation in one's area of residency, and the risk of infection with COVID-19 all contribute to the vaccination rate. At present, some parts of mainland China including Nanjing, Zhengzhou, and other places, are experiencing point-like outbreaks and the recurrence of the epidemic, a situation that encourage people to take COVID-19 vaccine. Similarly, we discovered that individuals' perceptions of the COVID-19 vaccine have an effect on vaccination uptake. The belief in the efficacy of vaccines and the necessity for universal vaccination promotes COVID-19 vaccination, which is consistent with earlier research (4, 13, 16). These findings underscore the critical importance of public education regarding the COVID-19 vaccine, which has demonstrated the potential to be exceedingly effective.

Certain background information had a minor but significant effect on the COVID-19 vaccination rate, which was also unaccounted for in previous studies. The results demonstrate that convenience to vaccination, having similar views as other residents on COVID-19 vaccination, being willing to discuss about COVID-19 vaccine-related issues with other residents, set up a special COVID-19 vaccination site, the outstanding administrative performance of community leaders, residents care for each other, and having plans to live in their respective community for an extended period of time contributed to COVID-19 vaccination uptake. This indicates that effective management at the grassroots level may make a significant contribution to the development of an epidemic prevention and control system. At the same time, attitudes and behaviors toward vaccines in general affect COVID-19 vaccination uptake. General vaccines have long been considered important preventive measures against certain diseases (1, 31, 32), and COVID-19 vaccine has been used for <2 years since it was developed. People's perception of vaccines in general may greatly impact their vaccination uptake. The findings in Table 3 indicate that the more accurate one's understanding of vaccinations is, the less concerned one is about their safety and effectiveness, and the higher the vaccination uptake. This is consistent with previous studies in the UK and Turkey and other countries (16, 23).

People's decisions on whether or not to get the COVID-19 vaccine will be influenced by their level of skepticism. Previous research in the United Kingdom and Italy found that skepticism about the epidemic and the COVID-19 vaccine can cause people to delay or refuse to receive the vaccine (4, 21, 33). Additionally, we discovered that there is a high level of skepticism regarding the epidemic or the vaccine, resulting in a very low vaccination rate. Detailed results are presented in Table 4. In our research, mainland Chinese residents were generally less skeptical about the epidemic and COVID-19 vaccine. Detailed results are shown in Annexes 3, 4.

We also found that there is a considerable link between distrust among doctors and vaccine developers when it comes to COVID-19 vaccination. Ultimately, the willingness to be vaccinated is a matter of trust that the vaccine is necessary, it will work, and it is safe (16, 28). Public trust in healthcare professionals and vaccine developers has declined significantly as a result of occasional vaccine-related adverse events and growing instances of counterfeit vaccines (34). Therefore, this is a reminder that we need to build confidence in vaccines. On the one hand, vaccination service institutions should increase the number of vaccination medical staff and enhance the quality of vaccination service evaluation; on the other hand, we should accelerate the development of the vaccine industry's credit system, encourage enterprises to take the lead in vaccine production and distribution, and ensure the quality and safety of vaccine products.

The current study also investigated the reasons why participants were not vaccinated against COVID-19 (*n* = 3,170). In addition to personal ineligibility for vaccination (15%) and temporary lack of vaccine supply at the vaccination site (17%), 20% of unvaccinated participants were concerned about the safety or efficacy of the vaccine while 10% of them wanted more information about the vaccine before making their decision. This shows that the main reason for individuals not receiving COVID-19 vaccination is still uncertainty regarding the vaccine's safety and efficacy, which is consistent with findings from previous studies in Russia and other countries (35–37). Therefore, in the subsequent explanatory work, we must emphasize the importance of the prosocial benefits of the COVID-19 vaccine, as well as the necessity of being transparent about the data on the safety and efficacy in order to further dispel people's doubts about the COVID-19 vaccine and improve the vaccination rate. This may aid in raising the willingness to uptake the vaccine.

This is the first survey to examine the current state of COVID-19 vaccination among Chinese residents throughout 31 provinces on the Chinese mainland. From a broader viewpoint, this article examines the factors that contribute to the current COVID-19 vaccine coverage rate. Prior research concentrated primarily on special groups, biosafety technologies, and vaccination willingness, whereas our research delved deeper into community environmental factors, epidemic/vaccine perception, epidemic/vaccine skepticism, and trust on vaccine developers, among other factors. However, there are limitations to the survey that need to be addressed. Our study's shortcomings include its cross-sectional design, which precluded the establishment of a cause-and-effect relationship. Some potential confounding factors may have impacted the robustness of our results, such as vaccine's adverse reactions. Moreover, despite the fact that we used data from a large sample of the population drawn from 31 provinces, due to the epidemic, we were compelled to collect data *via* online questionnaires using the snowball sampling technique. Therefore, these research findings may differ from those estimated using probability sampling. Additionally, the factors influencing COVID-19 vaccine uptake found in the current study may be different for individuals without Internet access. In future, we intend to conduct a larger survey with a more representative sample, using detailed in-home interviews to continuously track and assess the changes and causes of COVID-19 vaccination rate, as well as conducting intervention studies in a small area to infer causal associations between specific factors and COVID-19 vaccination rate.

## Conclusions

Thirty-one provinces in mainland China currently have a relatively high rate of COVID-19 vaccination. To further increase the rate of COVID-19 vaccination, we need to remove barriers associated with the community context and enhance the accessibility of COVID-19 vaccine services. Additionally, by taking proactive and effective actions to address the reasons for non-vaccination against COVID-19, the construction of an epidemic prevention and control system will be considerably facilitated.

## Data Availability Statement

The original contributions presented in the study are included in the article/Supplementary Materials, further inquiries can be directed to the corresponding author/s.

## Ethics Statement

Ethics study protocol and online survey were approved by the Life Science Ethics Review Committee of the Zhengzhou University.

## Author Contributions

YM, JW, MM, MW, and JG: conceptualization. YM, BY, MM, QL, and WW: data curation. XF, JW, QL, and YM: formal analysis. MW and JG: funding acquisition. JW, YM, BY, MM, QL, LZ, and ZM: investigation. JW, YM, MM, and CT: methodology. JW and XF: project administration. JW, YM, and XF: resources. MM, LZ, and ZM: software. MM and JW: writing—original draft. YM, QL, CT, MW, and JG: writing—review editing. All authors contributed to the article and approved the submitted version.

## Funding

This work was supported by the National Social Science Fund of China (No. 21BGL222), the Collaborative Innovation Key Project of Zhengzhou (No. 20XTZX05015), 2021 Postgraduate Education Reform and Quality Improvement Project of Henan Province (No. YJS2021KC07), Zhengzhou University 2020 Key Project of Discipline Construction (No. XKZDQY202007), the Performance Evaluation of New Basic Public Health Service Projects in Henan Province (No. 2020130B) and Teaching Reform Project of Zhengzhou University (No. 2021-153).

## Author Disclaimer

The content is solely the responsibility of the authors and does not necessarily represent the official views of the government agencies and NGOs.

## Conflict of Interest

The authors declare that the research was conducted in the absence of any commercial or financial relationships that could be construed as a potential conflict of interest.

## Publisher's Note

All claims expressed in this article are solely those of the authors and do not necessarily represent those of their affiliated organizations, or those of the publisher, the editors and the reviewers. Any product that may be evaluated in this article, or claim that may be made by its manufacturer, is not guaranteed or endorsed by the publisher.
