# Supplementary material for: COVID-19 Vaccination Acceptance Among Chinese Population and Its Implications for the Pandemic: A National Cross-Sectional Study
Source: Front Public Health. 2022 Feb 8;10:796467. doi: 10.3389/fpubh.2022.796467 (PMC8860971; doi:10.3389/fpubh.2022.796467)
Supplement: Supplementary file 1 [file Data_Sheet_1.docx]

Annex 1 Sample size calculation formula


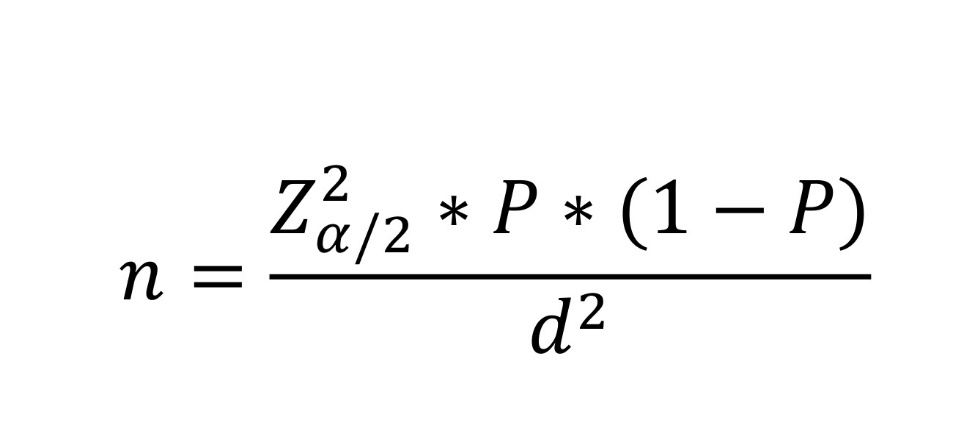


Note: The minimum sample size based on a prevalence of COVID-19 vaccination rate of 83.43% in the preliminary online survey, an allowable error of 1% and consider the missing 20% sample size.

Annex 2. Trend of prevalence rate of COVID-19 acceptance over sample saturation


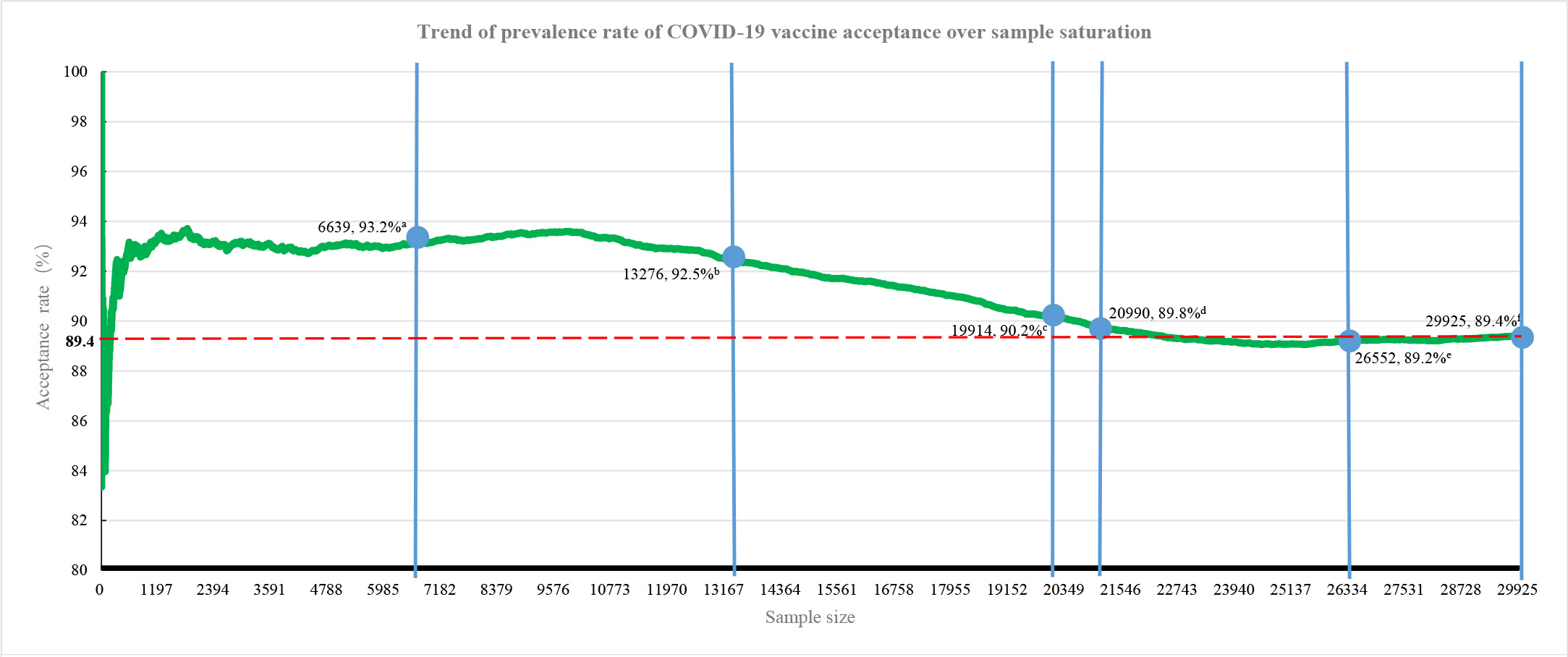


Note: The Chi-square goodness-of-fit test was used, and prevalence rate of COVID-19 vaccine acceptance was set at 89.4% in data perform process.

a, Chi-square=99.857, P<0.001; b, Chi-square=131.186, P<0.001; c, Chi-square=13.887, P<0.001; d, Chi-square=2.749, P=0.097; e, Chi-square=0.787, P=0.375; f, Chi-square=0.001, P=0.969.

Annex 3. Endorsement of coronavirus skepticism

|  | **Strongly agree** | **Agree** | [**Neutral**](javascript:;) | **Disagree** | **Strongly disagree** | **Do not know** |
| --- | --- | --- | --- | --- | --- | --- |
| **The virus is a hoax.** | 2655(8.9%) | 2103(7.0%) | 2307(7.7%) | 8014(26.8%) | 13592(45.4%) | 1254(4.2%) |
| **The virus is manmade.** | 3671(12.3%) | 4957(16.6%) | 4681(15.6%) | 5728(19.1%) | 7971(26.6%) | 2917(9.7%) |
| **The spread of the virus is a deliberate attempt to reduce the size of the global population.** | 2797(9.3%) | 2588(8.6%) | 3405(11.4%) | 7770(26.0%) | 11243(37.6%) | 2122(7.1%) |
| **The spread of the virus is a deliberate attempt by a group of powerful people to make money.** | 2879(9.6%) | 2610(8.7%) | 3504(11.7%) | 8008(26.8%) | 10568(35.3%) | 2356(7.9%) |
| **The spread of the virus is a deliberate attempt by governments to gain political control.** | 4016(13.4%) | 4483(15.0%) | 4160(13.9%) | 6056(20.2%) | 8494(28.4%) | 2716(9.1%) |
| **The spread of the virus is a deliberate attempt by one nation to destabilize another.** | 4228(14.1%) | 4831(16.1%) | 4510(15.1%) | 5752(19.2%) | 7810(26.1%) | 2794(9.3%) |
| **The spread of the virus is a deliberate attempt by global companies to take control.** | 3588(12.0%) | 3706(12.4%) | 4522(15.1%) | 6501(21.7%) | 8539(28.5%) | 3069(10.3%) |

Annex 4. COVID-19 vaccine skepticism

|  | **Strongly agree** | **Agree** | [**Neutral**](javascript:;) | **Disagree** | **Strongly disagree** | **Do not know** |
| --- | --- | --- | --- | --- | --- | --- |
| **The COVID-19 vaccine safety data is inaccurate.** | 2300(7.7%) | 2737(9.1%) | 5371(17.9%) | 9133(30.5%) | 8439(28.2%) | 1945(6.5%) |
| **The coronavirus vaccine is harmful, but the news is buried** | 2097(7.0%) | 2118(7.1%) | 3756(12.6%) | 9125(30.5%) | 11045(36.9%) | 1784(6.0%) |
| **Pharmaceutical companies cover up the dangers of vaccines.** | 2016(6.7%) | 2120(7.1%) | 4170(13.9%) | 9297(31.1%) | 10210(34.1%) | 2112(7.1%) |
| **People are deceived about the effectiveness of vaccines.** | 2112(7.1%) | 2064(6.9%) | 3952(13.2%) | 9424(31.5%) | 10348(34.6%) | 2025(6.8%) |
| **The COVID-19 vaccine effectiveness data is inaccurate.** | 1920(6.4%) | 2264(7.6%) | 4573(15.3%) | 9356(31.3%) | 9688(32.4%) | 2124(7.1%) |
| **People are deceived about vaccine safety.** | 2053(6.9%) | 2066(6.9%) | 3860(12.9%) | 9551(31.9%) | 10479(35.0%) | 1916(6.4%) |
| **Vaccination against COVID-19 may cause other diseases** | 1986(6.6%) | 2797(9.3%) | 5423(18.1%) | 8372(28.0%) | 8642(28.9%) | 2705(9.0%) |
